# The Development of New Nanocomposite Polytetrafluoroethylene/Fe_2_O_3_ NPs to Prevent Bacterial Contamination in Meat Industry

**DOI:** 10.3390/polym14224880

**Published:** 2022-11-12

**Authors:** Dmitriy A. Serov, Ilya V. Baimler, Dmitriy E. Burmistrov, Alexey S. Baryshev, Denis V. Yanykin, Maxim E. Astashev, Alexander V. Simakin, Sergey V. Gudkov

**Affiliations:** Prokhorov General Physics Institute of the Russian Academy of Sciences, 38 Vavilova St., 119991 Moscow, Russia

**Keywords:** iron oxide nanoparticles, food industry, Teflon, fluoroplast, antibacterial activity, cytotoxicity, reactive oxygen species

## Abstract

The bacterial contamination of cutting boards and other equipment in the meat processing industry is one of the key reasons for reducing the shelf life and consumer properties of products. There are two ways to solve this problem. The first option is to create coatings with increased strength in order to prevent the formation of micro damages that are favorable for bacterial growth. The second possibility is to create materials with antimicrobial properties. The use of polytetrafluoroethylene (PTFE) coatings with the addition of metal oxide nanoparticles will allow to the achieving of both strength and bacteriostatic effects at the same time. In the present study, a new coating based on PTFE and Fe_2_O_3_ nanoparticles was developed. Fe_2_O_3_ nanoparticles were synthesized by laser ablation in water and transferred into acetone using the developed procedures. An acetone-based colloidal solution was mixed with a PTFE-based varnish. Composites with concentrations of Fe_2_O_3_ nanoparticles from 0.001–0.1% were synthesized. We studied the effect of the obtained material on the generation of ROS (hydrogen peroxide and hydroxyl radicals), 8-oxoguanine, and long-lived active forms of proteins. It was found that PTFE did not affect the generation of all the studied compounds, and the addition of Fe_2_O_3_ nanoparticles increased the generation of H_2_O_2_ and hydroxyl radicals by up to 6 and 7 times, respectively. The generation of 8-oxoguanine and long-lived reactive protein species in the presence of PTFE/Fe_2_O_3_ NPs at 0.1% increased by 2 and 3 times, respectively. The bacteriostatic and cytotoxic effects of the developed material were studied. PTFE with the addition of Fe_2_O_3_ nanoparticles, at a concentration of 0.001% or more, inhibited the growth of *E. coli* by 2–5 times compared to the control or PTFE without NPs. At the same time, PTFE, even with the addition of 0.1% Fe_2_O_3_ nanoparticles, did not significantly impact the survival of eukaryotic cells. It was assumed that the resulting composite material could be used to cover cutting boards and other polymeric surfaces in the meat processing industry.

## 1. Introduction

Meat deboning is one of the stages of raw meat processing, during which muscle, connective, and adipose tissue, that is, meat itself, is separated from the bone content. Deboning is performed by hand or with special equipment, while the meat is usually on cutting boards during deboning. During the use of cutting boards, microdamages accumulate on the surface of the cutting boards [1]. In these microdamages, favorable conditions are created for the growth and development of pathogenic microorganisms, including the formation of bacterial communities. The development of bacteria on cutting boards leads to biological contamination of meat products and a decrease in its shelf life and deterioration of its organoleptic properties [2]. Unfortunately, antibiotic-resistant strains are often found among these bacterial strains [3,4]. Gastrointestinal infections are an important global health problem [5]. Over 70,000 cases of hospitalizations with severe intestinal infections are predicted annually in the United States alone, of which over 1800 cases are fatal [6]. There are two principal approaches in order to protect the surface of cutting boards from bacterial contamination. The first approach is the creation of self-healing and/or ultra-strong materials to prevent the formation of microdamages favorable for bacterial growth. There are data in the literature on the dependence of the degree of bacterial contamination of *S. enterica* on the cutting board material. In the case of using a more durable material (glass), bacterial contamination occurred in 10% cases, and in the case of a softer material, i.e., plastic or wood, bacterial growth was observed in 40 and 60% of cases [4]. The second approach is to create a material with antibacterial action to suppress the growth of already-attached microbes.

Polytetrafluoroethylene (PTFE, Teflon, fluoroplast) is a fluoropolymer consisting of tetrafluoroethylene monomers described with the formula …–(CF_2_-CF_2_)n-… [7]. PTFE has a number of interesting properties such as high thermal conductivity and mechanical strength, dielectric properties, a resistance to a wide range of chemical agents, etc., [8,9]. These properties make PTFE an ideal material for a wide variety of applications such as mechanical engineering, the electronic and chemical industries, the space industry, the pharmaceutical industry, the manufacturing of implants, air and water filtration and the meat processing industry [10,11,12,13,14,15,16,17,18]. PTFE has pronounced hydrophobic properties, the ability to self-lubricate, and is self-healing. These properties make it possible to create self-healing coatings based on PTFE [19,20,21]. It was assumed in this study that the use of PTFE coatings would increase the mechanical strength of cutting boards and solve the problem of microdamages. The use of nanoparticles of metals and their oxides is one of the promising ways to combat microorganisms, including those resistant to antibiotics [22]. Iron oxide nanoparticles have high toxicity against bacteria and relatively low cytotoxicity against animal and human cells [23].

Previously, combined materials based on metal oxide NPs, including Fe_2_O_3_ and polymer matrices using the example of borosiloxane and PLGA, were obtained [24,25,26]. These materials have shown excellent antibacterial properties. Due to its well-known bioinertness and lack of biodegradability, fluoroplast is a good candidate for a matrix for the manufacture of stable antibacterial composite materials. A fundamental possibility for creating materials based on PTFE and metal oxide NPs using Ag_2_O as an example was described earlier [27]. Fe_2_O_3_ was chosen in this study because it is a common iron oxide, and it is cheaper to obtain. A polymer varnish was chosen as a PTFE-containing base. It was assumed that it would completely fill in the irregularities of the working surface and existing microdamages. An important requirement is the smoothness of the surface after processing.

Summarizing the above, the aim of this study was to obtain a new composite coating based on PTFE and Fe_2_O_3_ nanoparticles (PTFE/Fe_2_O_3_ NPs) and to study its physical properties, surface features, its influence on the generation of reactive oxygen species, namely 8-oxoguanine in DNA and long-lived reactive protein species (LRPS), its bacteriostatic activity, and its cytotoxicity against eukaryotic cells. 

For the first time, a composite coating based on PTFE polymer and Fe_2_O_3_ NPs was obtained, and its high adhesion to surfaces made of damaged fluoroplastic was shown. We found for the first time that the PTFE/Fe_2_O_3_ NP composite coating caused an increase in the generation of ROS, 8-oxoguanine in DNA in vitro, and LRPS, that it had a strong bacteriostatic effect, and did not affect the number of viable fibroblasts in the primary culture.

## 2. Materials and Methods

### 2.1. Preparation of Composite PTFE/Fe_2_O_3_ NPs

Fe_2_O_3_ nanoparticles were obtained by laser ablation in water using a Nd:YAG laser [28]. Chemically pure iron Fe 99.9% (Sigma Aldrich, Burlington, MA, USA) was used as a target. The resulting Fe_2_O_3_ nanoparticles were precipitated three times with centrifugation followed by dilution in acetone. The resulting Fe_2_O_3_ NPs colloid in acetone was mixed with a LF-32LN PTFE-based varnish, 3V-00001283 (Plastpolymer-Prom, St. Petersburg, Russia). The mixture was stored in glass jars until use. Fe_2_O_3_ NPs were added at concentrations of 0.001, 0.01, or 0.1% (m/m).

To study the possibility of leveling damage and adhesion to the surface, we applied a composite coating to the area of the fluoroplast sample damaged during operation. The coating was dried for 48 h. The area of damaged fluoroplasts was photographed before and after coating.

In experiments on the effect of PTFE/Fe_2_O_3_ NPs composite material on the generation of ROS, the formation of 8-oxoguanine in DNA, the formation of LRPS, as well as evaluating its bacteriostatic effect, coatings containing Fe_2_O_3_ NPs at concentrations of 0.001–0.1% or PTFE without NPs were used. To study the cytotoxicity of the coating in relation to primary cultures of fibroblasts, the maximum of the considered concentrations of Fe_2_O_3_ NPs (0.1%) was used. Coatings (V = 500 μL) were applied to the surfaces of round coverslip glasses with a diameter of 25 mm and dried for 48 h in a fume hood. The final thickness of the dried coating was no more than 200 μm. For biological studies, samples of coatings on glasses were sterilized by soaking in 70% ethanol for 3 h. For microbiological studies, samples of coatings were removed from glasses in the form of films with an area of ~20 cm^2^. To study cytotoxicity, coating samples were dried on glass slides under sterile conditions.

### 2.2. Physical Properties Assay

The main characteristics of the obtained nanoparticles were studied, namely their size, ζ-potential, absorption spectrum, and shape. The size was estimated using DLS methods with Zetasizer Ultra Red Label (Malvern Panalytical, Malvern, Worcestershire, UK) and TEM with a Libra 200 FE HR electron microscope (Carl Zeiss, Oberkochen, Germany). ζ-potential was measured using Zetasizer Ultra Red Label (Malvern Panalytical, Malvern, Worcestershire, UK). Absorption spectra in the UV–visible region were recorded using a Cintra 4040 spectrometer (GBC Scientific Equipment, Braeside, VIC, Australia). The surface structure of the PTFE/Fe_2_O_3_ NPs composite was examined using atomic force microscopy (AFM) using SII Nanopics 2100 (KLA-Tencor, Milpitas, CA, USA). The distribution of Fe_2_O_3_ nanoparticles inside the composite was evaluated with modulation interference microscopy (MIM) using MIM-32 (Amphora Lab, Korolyov, Moscow region, Russia). A more detailed description of research methods can be found in earlier research studies [26,29,30].

### 2.3. Measurement of Reactive Oxygen Species Concentration

The chemiluminescent method of the luminol–*p*-iodophenol–horseradish peroxidase system was used to estimate the concentration of formed H_2_O_2_. Chemiluminescence was recorded using a highly sensitive chemiluminometer, Biotoks-7A (Engineering Center—Ecology, Moscow, Russia). The calibration and registration procedure was carried out according to the protocols described in more detail earlier. Films with an area of 20 cm^2^ from a composite material containing various concentrations of Fe_2_O_3_ NPs in compositions from 0–0.1 wt.% were placed in polypropylene vials for 3 h at 40 °C. In the “Control” group, the experiment was carried out without coating sample. After incubation in 20 mL of water, 1 mL of a previously prepared “counting solution” was added to the sample. This solution contained 1 mM Tris–HCl buffer, pH 8.5, 50 µM p-iodophenol, 50 µM luminol, and 10 nM horseradish peroxidase. The sensitivity of this method made it possible to determine H_2_O_2_ at a concentration of <1 nM. [31] The concentration of OH radicals formed in aqueous solutions was determined by reaction with coumarin-3-carboxylic acid (CCA), the product of which was hydroxycoumarin-3-carboxylic acid (7-OH-CCA). 7-OH-CCA is a fluorescent probe that quantifies OH radicals. Briefly, 0.2 M PBS (pH 7.4) was added to a solution of CCA in water (0.5 mM, pH 3.6). Next, samples of the composite material containing various concentrations of Fe_2_O_3_ NPs in compositions from 0–0.1 wt.% were added to the vials. In the “Control” group, the experiment was carried out without a coating sample. Next, polypropylene vials with samples and reagents were heated in a thermostat at temperature of 80.0 ± 0.1 °C for 2 h. Fluorescence of 7-OH-CCA was recorded using a JASCO 8300 fluorimeter (JASCO, Hachioji, Japan) at λ_ex_ = 400 nm (excitation wavelength) and λ_em_ = 450 nm (emission wavelength). Commercial 7-OH-CCA (Sigma-Aldrich, Burlington, MA, USA) was used for calibration [32].

### 2.4. Measurement of 8-Oxoguanine and Long-Lived Reactive Protein Species Concentration

A non-competitive enzyme-linked immunosorbent assay (ELISA) was used to quantify 8-oxoguanine in DNA using monoclonal antibodies specific for 8-oxoguanine (anti-8-OG antibodies). DNA samples (350 μg/mL) were denatured by boiling in a water bath for 5 min and subsequently cooled on ice for 3–4 min. Aliquots (42 μL) were applied to the bottom of the wells of the ELISA plates. The DNA was immobilized using a simple adsorption procedure with incubation for 3 h at 80 °C until the solution became completely dry. Non-specific adsorption sites were blocked with 300 μL of a solution containing 1% skimmed milk powder in 0.15 M Tris-HCl buffer, with pH 8.7 and 0.15 M NaCl. Next, the plates were incubated at room temperature overnight (14–18 h). The formation of an antigen–antibody complex with antibodies against ti-8-OG (at a dilution of 1:2000) was carried out in a blocking solution (100 µL/well) by incubation for 3 h at 37 °C. The samples were washed twice (300 µL/well) with 50 mM Tris–HCl buffer (pH 8.7) and 0.15 M NaCl with 0.1% Triton X-100 after 20 min incubation. Next, a complex with a conjugate (anti-mouse immunoglobulin labeled with horseradish peroxidase (1:1000)) was formed by incubation for 1.5 h at 37 °C in a blocking solution (80 µL/well). Then, the wells were washed 3 times as described above. Next, a chromogenic substrate containing 18.2 mM ABTS and hydrogen peroxide (2.6 mM) in 75 mM citrate buffer, pH 4.2 (100 µL/well), was added to each well. Reactions were stopped by adding an equal volume of 1.5 mM NaN_3_ in 0.1 M citrate buffer (pH 4.3) upon reaching the correct color. The optical density of the samples was measured on a plate photometer Titertek Multiscan (Flow laboratories, McLean, VA, USA) at λ = 405 nm.

The chemiluminescent method is effective and sensitive for determining free radical reactions. The interaction of radicals is accompanied by the release of energy in the form of emitted light quanta [33]. In this case, the interaction of radicals releases energy, which is emitted in the form of light quanta. Chemiluminescence was recorded using a highly sensitive chemiluminometer, Biotoks-7A (Engineering Center—Ecology, Moscow, Russia). The samples were heated to a temperature of 45 °C within 2 h. Measurements were carried out in the dark at room temperature in 20 mL plastic polypropylene vials (Beckman, Brea, CA, USA). All samples were stored in the dark at room temperature for 30 min after exposure. As a control, protein solutions without preheating were used. A more detailed description of the method is presented in the published article by Sharapov et al. [34].

### 2.5. Antimicrobial Avtivity Assay

The antibacterial effect of composite films with an area of ~20 mm^2^ with different concentrations of Fe_2_O_3_ NPs was studied against the Gram-negative bacterium Escherichia coli. Samples were pre-styled by incubation in 70% ethanol for 3 h. Next, each sample was placed in a sterile wrap with 5 mL of liquid LB nutrient medium (LenReaktiv, Moscow, Russia). A known equal number of *E. coli*-colony-forming units (CFU) was added to each hoop with or without an image (control). The number of CFUs was pre-determined with a counting chamber and these were used for further calibration. Hoops with samples and LB and *E. coli* medium were covered with film and incubated for 24 h at 37 °C, ~150 rpm in an incubator shaker (Biosan, Riga, Latvia). The number of bacteria was estimated from the optical density at 600 nm of the suspension using a UV5Nano Excellence drop spectrometer (Mettler Toledo, Columbus, OH, USA). The value of the measured optical density was recalculated into the number of CFUs per unit volume using the previously constructed calibration [25,35,36].

### 2.6. Assay of the Influence on Animal Cells Viability

The study was performed on a primary culture of murine fibroblasts (BALB/c males weighing 22–25 g), which were isolated and cultivated according to a standard protocol [37]. All experiments with laboratory animals were carried out in accordance with the regulatory legal act of the Ministry of Health of the Russian Federation №199-n “On approval of the rules of good laboratory practice” and the international legal norms specified in the European Convention ETS №123 “On the protection of vertebrate animals used for experiments or for other scientific purposes”. Round coverslips were coated by PTFE with the addition of 0.1% Fe_2_O_3_ nanoparticles or PTFE without nanoparticles and were dried for 24 h in a fume hood. Cells were seeded on prepared slides and uncoated slides (control) and cultured in DMED/F12 medium supplemented with 10% FBS, 2 mM L-glutamine, and antibiotics (Sigma Aldrich, Burlington, MA, USA) for 72 h at 37 °C and 5% CO_2_. After incubation, cells were stained with fluorescent dyes Hoechst33342 and PI (Sigma Aldrich, Burlington, MA, USA) and were analyzed using a DMI6000 fluorescent microscope (Leica, Wetzlar, Germany) [38,39,40].

### 2.7. Statistic

All data are presented as means ± standard errors. Statistical significance was assessed using the Mann–Whitney test. In each variant, the experiments were performed with at least three independent repetitions.

## 3. Results

### 3.1. Physical Properties

**Figure 1:**
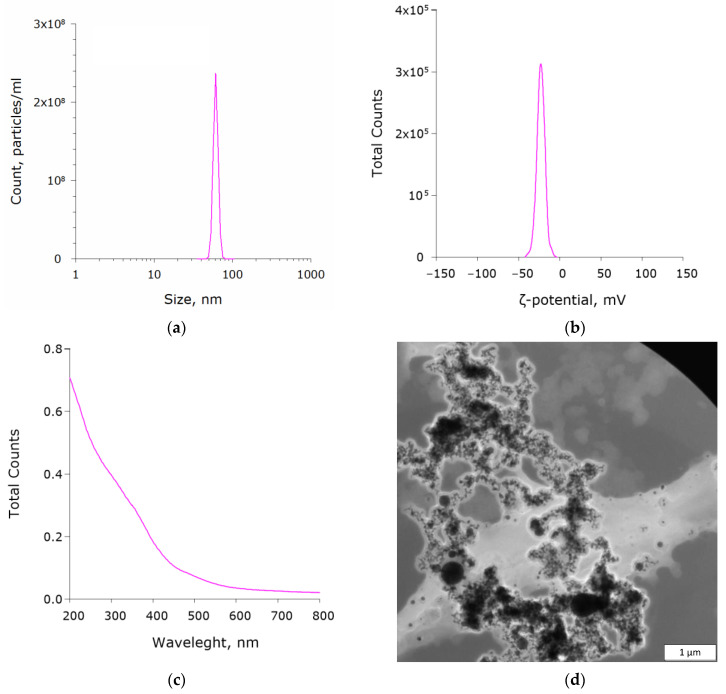
Physical characteristics of Fe_2_O_3_ NPs: (**a**) size distribution histogram; (**b**) ζ-potential distribution histogram; (**c**) UV–visible absorbance spectrum; (**d**) TEM image (scale bar—1 μm).

According to the DLS data, the size distribution of the synthesized Fe_2_O_3_ nanoparticles was symmetrical. The size ranged from 43 to 87 nm with an average value of ~61 nm (Figure 1a). The ζ-potential values ranged from −69 to −7 mV with a mean value of −25 mV at colloid pH 6.7 ± 0.1 (Figure 1b). The absorption spectrum clearly showed absorption at wavelengths <400 nm, which was characteristic of Fe_2_O_3_ nanoparticles (Figure 1c). According to the electron microscopy, the size distribution of the nanoparticles was more heterogeneous, and the shape was spherical (Figure 1d). Virtually no aggregates of nanoparticles were found. 

**Figure 2:**
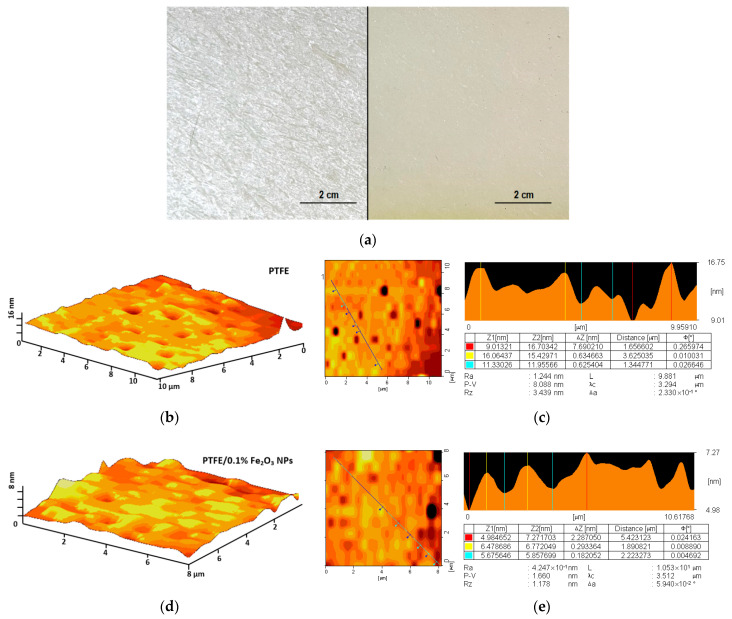
Example of applying a PTFE-based coating to a damaged surface of a fluoroplast before (left) and after (right) coating (**a**); AFM images of composite material surface: (**b**) 3D reconstruction of PTFE without NPs; (**c**) result of analysis of PTFE selected area (shown as a diagonal line in the figure) without NPs surface; (**d**) 3D reconstruction of PTFE/0.1% Fe_2_O_3_ NPs; (**e**) result of analysis of PTFE/0.1% Fe_2_O_3_ NPs surface. The results of the analysis in panels (**c**) and (**e**) are presented in the form of profiles and a table with data on the depth of the asperities in the sample.

We found that the composite coating perfectly adhered to the damaged surface of the PTFE, covering all visible damage (Figure 2a). When trying to mechanically damage the surface 48 h after coating, there were no visible violations of the integrity of the coating and the underlying layer. At the next stage of this research, the surface of the composite material was studied using the AFM method, and the distribution of nanoparticles inside the polymer matrix was estimated. The atomic force microscopy of the PTFE surface without the addition of nanoparticles showed the absence of irregularities and inhomogeneities (Figure 2b). The change in the relief of the PTFE surface did not exceed 10 nm (Figure 2c). The surface of the PTFE/0.1% Fe_2_O_3_ composite material was also free from cracks, wrinkles, and other damages and defects. (Figure 2d). The change in the surface relief of PTFE/0.1% Fe_2_O_3_ also did not exceed 10 nm (Figure 2e).

**Figure 3:**
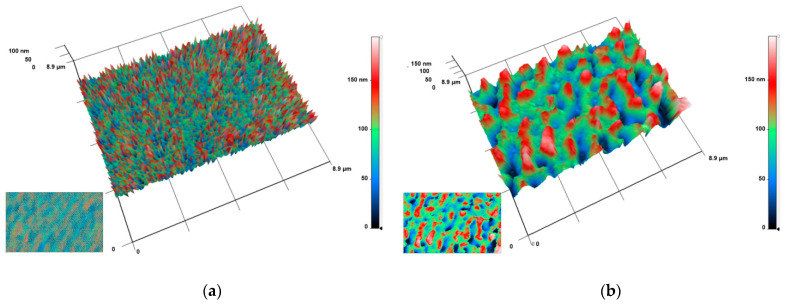

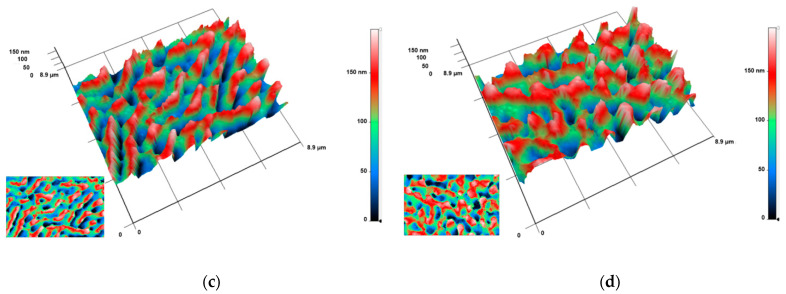
The results of the analysis of the obtained materials by MIM method: (**a**) PTFE without the addition of nanoparticles; (**b**) PTFE with the addition of 0.001% Fe_2_O_3_ nanoparticles; (**c**) PTFE with the addition of 0.01% Fe_2_O_3_ nanoparticles; (**d**) PTFE with the addition of 0.1% Fe_2_O_3_ nanoparticles. The images are presented as 3D reconstructions, where the abscissa and ordinate axes correspond to the real distance in μm. The Oz axis displays the phase difference in nm (the larger the phase difference, the higher the value on the Oz axis). Coloring is a pseudo-color. The initial data on the spatial distribution of the phase difference in the analyzed sample, used to construct 3D reconstructions, are shown in the lower left corners of each panel.

The distribution of nanoparticles inside the composite material was studied using the modulation-interference microscopy method, which made it possible to distinguish the objects with different values of the refractive index. In examining the thickness of PTFE without the addition of NPs, no pronounced inhomogeneities were found (Figure 3a). When Fe_2_O_3_ nanoparticles were added to PTFE at a concentration of 0.001%, inhomogeneities were found in the sample (Figure 3b). These inhomogeneities had a length of 0.25–1.0 μm and a width of 0.20–0.25 μm. The largest value of the phase difference was 150–160 nm. In the case of the PTFE composites with Fe_2_O_3_ nanoparticles at concentrations of 0.01 and 0.1%, a similar pattern was observed (Figure 3c,d), though the size of the inhomogeneities increased with the increase in the nanoparticle concentration. At a concentration of Fe_2_O_3_ nanoparticles of 0.01%, the length and width of the inhomogeneities were 0.5–2 and 0.2–0.25 μm, respectively. The phase difference was 150–170 nm (Figure 3c). At the maximum concentration of Fe_2_O_3_ nanoparticles of 0.1%, the length, width, and phase difference of the inhomogeneities were 0.5–2, 0.25–0.5 μm, and 150–170 nm, respectively (Figure 3d).

### 3.2. Generation of Reactive Oxigen Species Concentration

**Figure 4:**
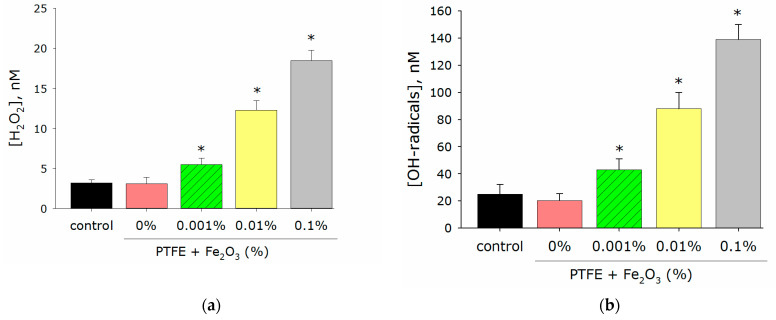
Effect of PTFE/Fe_2_O_3_ NPs on generation of free radicals: (**a**) hydrogen peroxide (2 h, 40 °C); (**b**) OH radials (2 h, 80 °C). Data are presented as means ± standard errors. *—*p* < 0.05 vs. control, Mann–Whitney test (*n* = 3).

An imbalance between the processes leading to the accumulation of ROS and the ability to remove the excess ROS due to antioxidant systems can lead to an increase in the concentration of ROS and a state of “oxidative stress”, which is dangerous for eukaryotic cells [41]. Therefore, it was very important that the developed materials did not cause excessive production of ROS. Considering the above, the effect of the PTFE/Fe_2_O_3_ NPs composite material on the generation of ROS was investigated using H_2_O_2_ and hydroxyl radicals as an example (Figure 4b). The concentration of H_2_O_2_ in the case of PTFE without nanoparticles did not differ from the control (Figure 4a). A variant of the PTFE/Fe_2_O_3_ NPs composite material with a nanoparticle concentration of 0.001% increased the H_2_O_2_ concentration by almost 2 times compared to the control. In the presence of PTFE/Fe_2_O_3_ NPs composites with nanoparticle concentrations of 0.01% and 0.1%, an increase in the H_2_O_2_ concentration by 4 and 6 times, respectively, was recorded. The PTFE polymer without nanoparticles did not change the concentration of hydroxyl radicals compared to the control (Figure 4b). In the presence of a PTFE/Fe_2_O_3_ NPs composite with nanoparticle concentrations of 0.001, 0.01, and 0.1%, an increase in the concentration of hydroxyl radicals by 2, 4, and 7 times, respectively, was observed.

### 3.3. Generation of 8-Oxoguanine and Long-Lived Reactive Protein Species

At the next stage of the study, the effect of the developed composite material on the generation of markers of oxidative damage to the biological polymers was evaluated. In the case of DNA, this was 8-oxoguanine; in the case of proteins, it was long-lived reactive protein species (LRPS).

**Figure 5:**
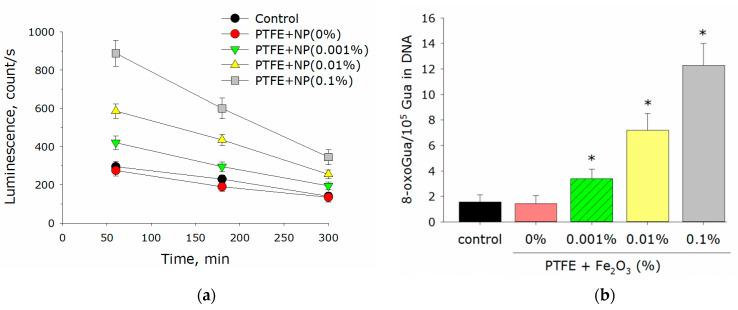
Effect of PTFE/Fe_2_O_3_ NPs on generation of free radicals: (**a**) LRPS (2 h, 40 °C); (**b**) 8-oxoguanine (8-oxoGua) in DNA in vitro (2 h, 45 °C). Data are presented as means ± standard errors. *—*p* < 0.05 vs. control, Mann–Whitney test (*n* = 3).

The PTFE coating without nanoparticles did not change the production of LRPS and 8-oxoguanine compared to control (Figure 5a,b). The PTFE composite with a concentration of Fe_2_O_3_ nanoparticles of 0.001% increased the concentration of LRPS by 25% compared to the control. The addition of Fe_2_O_3_ nanoparticles at a concentration of 0.01 and 0.1% to the composite material increased the generation of LRPS by 2 and 3 times, respectively, compared to the control (Figure 5a). The addition of 0.001% Fe_2_O_3_ nanoparticles to the composite material doubled the concentration of 8-oxoguanine compared to the control. PTFE/Fe_2_O_3_ composites with nanoparticle concentrations of 0.01 and 0.1% Fe_2_O_3_ NPs increased the production of 8-oxoguanine by 4 and 6 times compared to the control, respectively (Figure 5b).

### 3.4. Antimicrobial Activity

**Figure 6:**
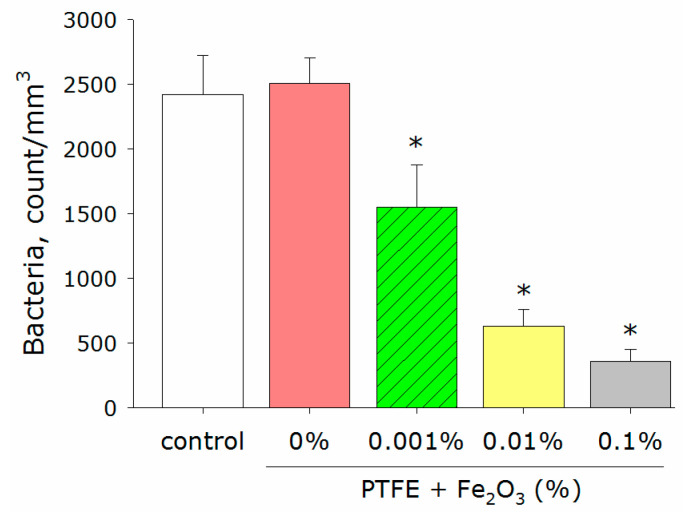
Examination of antibacterial activity of composite PTFE/Fe_2_O_3_ NPs against *E. coli*. Data are presented as means ± standard errors. *—*p* < 0.05 vs. control, Mann–Whitney test (*n* = 3).

The PTFE polymer without the addition of nanoparticles did not affect the bacterial growth rate compared to the control (Figure 6). The addition of Fe_2_O_3_ NPs to the PTFE significantly reduced the number of bacteria in the sample. The effect was dose-dependent. PTFE with a Fe_2_O_3_ NP concentration of 0.001% reduced the number of bacteria by ~40%, whereas the PTFE with an Fe_2_O_3_ NP concentration of 0.1% reduced the number of bacteria by more than 85% compared to the control.

### 3.5. Influence on the Animal Cells Viability

**Figure 7:**
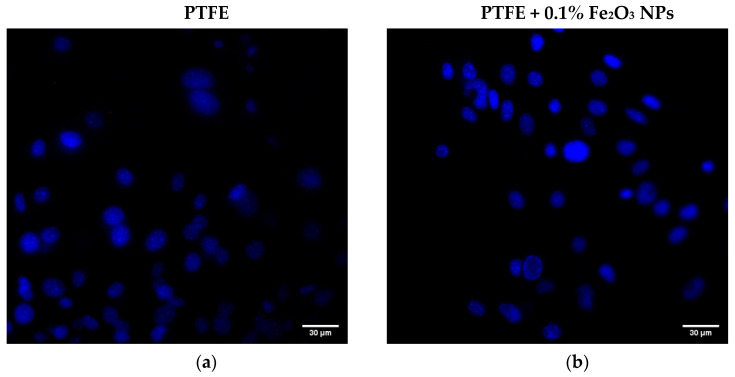

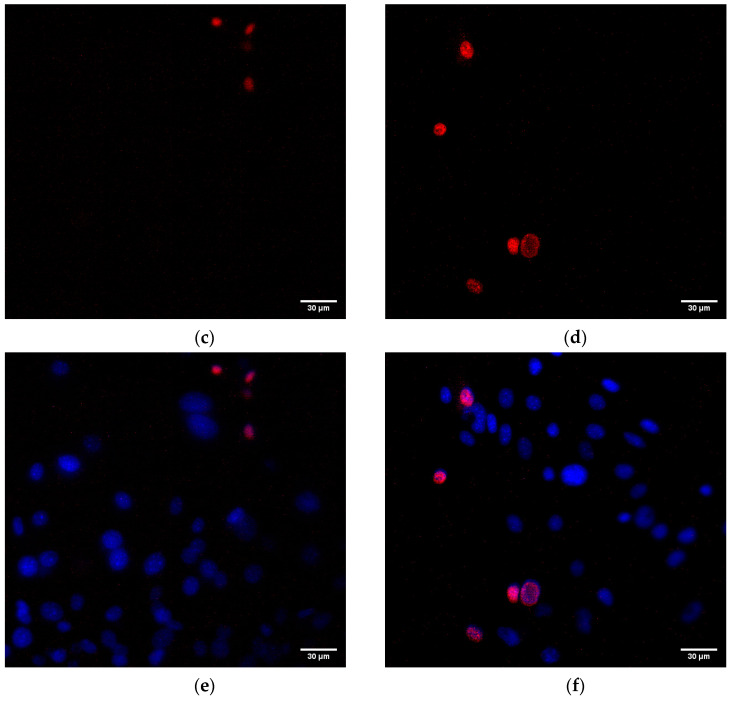
Examples of fluorescent microscopy images of cells during cytotoxicity study: (**a**) Hoechst (blue stain) image of cells on PTFE; (**b**) Hoechst image of cells on PTFE + 0.1% Fe_2_O_3_ NPs; (**c**) PI (red stain) of cells on PTFE; (**d**) PI of cells on PTFE + 0.1% Fe_2_O_3_ NPs; (**e**) merged image of cells on PTFE; (**f**) merged image of cells on PTFE + 0.1% Fe_2_O_3_ NPs. Scale bar is 30 μm. Magnification ×200, image resolution 1024 × 1024 pxl^2^.

At the last stage of this research, the effect of the obtained coatings on the viability of animal cells was studied using murine fibroblasts as an example. As criteria for evaluation, the proportion of dead cells and the size of the nucleus were chosen. The proportion of dead cells was defined as the ratio of the number of dead cells whose nuclei were stained with PI (Figure 7c,d) to the total number of cells whose nuclei were stained with Hoechst33342 only (Figure 7a,b).

**Figure 8:**
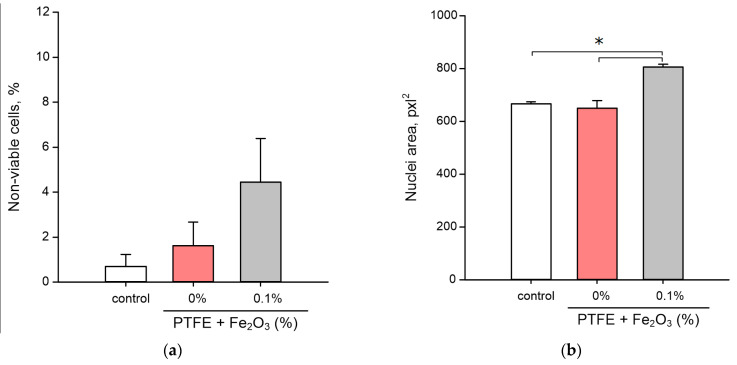
Cytotoxicity of composite PTFE/Fe_2_O_3_ NPs: (**a**) percentage of non-viable cells, where the amount of all analyzed cells was taken as 100%; (**b**) average nuclei size of cells. Data are presented as means ± standard errors. *—*p* < 0.05, Mann–Whitney test (*n* = 3).

Examples of staining with both dyes are shown in Figure 7e,f. The number of dead cells in the control was 0.7 ± 0.5% (Figure 8a). The average value of the proportion of dead cells on the PTFE without nanoparticles was 1.8 ± 1.0%, however, statistically significant differences in the proportion of dead cells between the PTFE and control were not found. The proportion of dead cells on the PTFE/Fe_2_O_3_ NPs 0.1% composite was 4.5 ± 1.9%. Despite the significant difference in the mean values, statistical significance between the proportions of dead cells during cultivation on PTFE/Fe_2_O_3_ NPs 0.1%, PTFE without nanoparticles, and/or the control was not found.

The mean nuclear area in the control was 665 ± 7 pxl^2^ (Figure 8b). When cultured on PTFE without nanoparticles, the cell nuclei had a size comparable to the control, i.e., 650 ± 28 pxl^2^. During cultivation on the PTFE/Fe_2_O_3_ NPs 0.1% composite, an increase in the average nucleus area up to 806 ± 11 pxl^2^ was found, which was statistically different from the control and cultivation on PTFE.

## 4. Discussion

The aim of this study was to obtain a new composite material of PTFE/Fe_2_O_3_ NPs, to study its physical properties and surface features, its influence on the generation of reactive oxygen species, 8-oxoguanine in DNA, and long-lived active protein species (LRPS), its bacteriostatic activity, and its cytotoxicity against eukaryotic cells.

In earlier studies, materials based on different polymer matrices (borosiloxane and PLGA) and nanoparticles of ZnO and Ag_2_O oxides were obtained [35,42]. In the present study, we developed a new composite coating based on PTFE and Fe_2_O_3_ NPs. Fe_2_O_3_ nanoparticles for the fabrication of the material were obtained by laser ablation in water. The resulting nanoparticles were about 60 nm in size (Figure 1a) and were spherical in shape (Figure 1d). These data were consistent with the literature data [43,44]. The ζ-potential values of the synthesized nanoparticles ranged from −69 to −7 mV with an average value of −25 mV at pH 6.7 ± 0.1 (Figure 1b). The obtained values of the ζ-potential indicated the satisfactory stability of the aqueous colloids synthesized with Fe_2_O_3_ NPs [45].

After obtaining the PTFE/Fe_2_O_3_ NP composite, its surface was characterized by the AFM method (Figure 2), and the distribution of NPs in the thickness of the polymer matrix was also estimated (Figure 3). On the surface of the PTFE without NPs, noticeable microdamages or defects (cracks, scales, etc.) were not found (Figure 2a,b). The surface of the PTFE/Fe_2_O_3_ NP composite was also smooth and free from microdamages (Figure 2c,d). Using the MIM method, it was found that the nanoparticles formed aggregates or clusters in the thickness of the polymer matrix of the PTFE/Fe_2_O_3_ NP composite (Figure 3b–d), which was consistent with data obtained in the earlier studies of the research team on polymer composites with nanoparticles of oxides of other metals [36].

There are several ways to additionally increase the strength of PTFE coatings such as adding selenium microparticles to the polymer matrix or creating composites with complex compositions, such as polyoxymethylene/glass fiber/polytetrafluoroethylene and polytetrafluoroethylene/polysulfone [46,47,48]. The inclusion of graphene, poly-*p*-phenyleneterephthalamide fibers, or glass fibers in the PTFE matrix makes it possible to modify the mechanical properties of PTFE (impact and tensile strength, hardness) [49]. The possibility of increasing the strength of composites of epoxy resins and PTFE by adding TiO_2_ powder to the matrix has been reported [50]. The search for methods to improve the physical properties of the developed composites is a task of further research.

The formation of moderate amounts of ROS normally occurs in any aerobic cell. In addition, ROS play an important role in the regulation of proliferation, differentiation, and migration of eukaryotic cells [51]. Excess ROS is normally removed during the functioning of antioxidant systems. Excessive generation of ROS, often caused by external causes, can lead to a state of “oxidative stress” that is dangerous for the integrity and functioning of cells [23,52,53]. At the cellular level, it can lead to mutagenesis, protein inactivation, lipid peroxidation, etc., [54]. At the level of the organism, “oxidative stress” is associated with carcinogenesis, genotoxic effects, and aging [41]. An important role in triggering “oxidative stress” is played by hydrogen peroxide (the most stable ROS) and hydroxyl radicals (the most reactive ROS) [31,55,56]. In addition to ROS, there are other inducers and markers of “oxidative stress” such as 8-oxoguanine, which is formed during the oxidative modification of DNA, and LRPS, which are formed during protein modification. LRPS themselves can be sources of new secondary radicals [57]. Considering all this, the ability of the developed PTFE/Fe_2_O_3_ NPs composite material to influence the generation of ROS using the examples of H_2_O_2_ and hydroxyl radicals, 8-oxoguanine, and LRPS was tested. It was found that the PTFE did not affect the generation of all the tested compounds (Figure 4 and Figure 5). The composite material of PTFE/Fe_2_O_3_ NPs increased ROS generation compared to the control (Figure 4 and Figure 5). However, the amount of ROS generated was negligible and should not cause oxidative stress [58].

The coating based on PTFE/Fe_2_O_3_ NPs showed a significant bacteriostatic effect (Figure 6) at a Fe_2_O_3_ NPs concentration of 0.1%, which corresponded approximately to 10 µg/mL. It was found that the PTFE without nanoparticles did not affect bacterial growth. Therefore, the bacteriostatic effect was provided by the Fe_2_O_3_ NPs. Several mechanisms of the antibacterial action of metal oxide nanoparticles have been described in the literature. The first mechanism is the direct disruption of the integrity of bacterial cell walls [59,60,61,62,63]. The second mechanism is the realizing of metal ions (in our case Fe^2+^), which bind to the SH groups of bacterial proteins. This leads to protein inactivation, an imbalance between the formation of ROS and the functioning of antioxidant systems, which can cause "oxidative stress", and the disruption of the functioning of ATPases [22,64,65,66,67,68,69,70]. The third mechanism is the generation of ROS as a result of the Fenton reaction or similar reactions with the participation of Fe^2+^ cations released from NPs [71,72]. The fourth mechanism is photocatalysis or the enhancement of ROS generation in the presence of light [73]. The fifth mechanism is the inhibition of DNA replication and genotoxic effects [48,74]. The sixth mechanism is unique for the NPs of some iron oxides with magnetic properties. When an external alternating magnetic field is applied, iron oxide NPs can cause the heating and dissociation of bacterial biofilms and the destruction of individual cells [75,76]. However, these magnetic properties are characteristic of Fe_3_O_4_; therefore, in our case, the implementation of this mechanism was extremely unlikely. Using the AFM and MIM methods, we showed that NPs were distributed in the thickness of the polymer matrix (Figure 2 and Figure 3). Based on this, we could assume that the probable mechanisms of the bacteriostatic action of the Fe_2_O_3_ NPs in our case were those that are due to the release of Fe^2+^ from NPs (the second and third mechanisms). However, the participation of the whole NPs in the bacteriostatic activity of the PTFE/Fe_2_O_3_ NPs composite coating could not be completely ruled out. It is described in the literature that the release of NP clusters from a polymer matrix is one of the mechanisms of the antibacterial action of nanocomposites [77]. It can be assumed that the prolonged bacteriostatic effects may have been associated with the gradual release of NPs or ions from the composite coating. It should be noted that the Fe_2_O_3_ could “protrude” from the polymer matrix in part of the NPs by an insignificant amount (less than 5–10 nm above the surface level, Figure 2), so the “contact” mechanism (first) of antibacterial activity could not be completely excluded. Clarification of the mechanisms of the antibacterial activity of PTFE/Fe_2_O_3_ NPs is a task for further research. An evaluation of the degree and duration of the release of Fe_2_O_3_ NPs from the polymer matrix during use, the dynamics of the generation of reactive species, and the antibacterial action of the released Fe_2_O_3_ NPs is planned to be studied in the future.

A comparison of these results with the data of earlier research is shown in Table 1. The antibacterial activity of iron oxide NPs depends on the degree of oxidation of iron in the oxide and the size of the NPs. NPs with large sizes exhibit lower antibacterial activity than smaller ones [78,79,80]. The nanoparticles obtained in these particular experiments had a size of about 50–70 nm and an MIC of 10 μg/mL, which was consistent with the literature data [79,81]. Fe_2_O_3_ NPs have a more pronounced antibacterial activity than Fe_3_O_4_ NPs [60,81,82]. The elemental composition of the nanoparticles obtained in this study by laser ablation in water was established by the research team earlier and corresponded to Fe_2_O_3_ [83]. These results were consistent with the data on the higher toxicity of Fe_2_O_3_ nanoparticles than Fe_3_O_4_ nanoparticles against bacteria. Combinations of Fe_2_O_3_ nanoparticles with nanoparticles of other metals and their oxides and polymer materials are considered as ways to improve antibacterial properties or reduce cytotoxicity against animal and human cells [84,85,86]. In this case, the antibacterial effect largely depends on the type of polymer used. For example, the addition of chitosan gives a more pronounced bacteriostatic effect than carbon nanotubes or antibiotics [85,86,87]. There are few published studies on the use of metal oxide nanoparticles in a PTFE-based matrix, and these are devoted to the study of the antimicrobial properties of Ag nanoparticles [27,88]. The concentrations of Ag nanoparticles at which the growth of microorganisms is inhibited (5–50 mg/mL) are, strictly speaking, the only ones used from the relevant works [27,88]; therefore, their MICs can be much lower than the indicated values. Previously, a composite material based on PLGA and Fe_2_O_3_ NPs was obtained. It also showed excellent antibacterial properties with an MIC of 10 μg/mL [83]. It is noteworthy that the MIC ~10 μm/mL did not depend on the type of polymer matrix (PLGA or PTFE) [83]. Based on the data of the present and earlier studies, it can be concluded that the inclusion of metal oxide nanoparticles inside the polymer matrix is a more effective way of improving the antimicrobial properties of materials than coating the individual nanoparticles with conjugates [27,83,86,87].

The literature describes other approaches to create antibacterial coatings for cutting boards with increased strength, in particular, the reconciliation of food-safe oil coatings [12,89]. These coatings make it possible to effectively fill microdamages and reduce the attachment of bacteria to the surface of cutting boards [12,89,90]. However, the application process of food-safe oil coatings is multi-stage. We made an attempt to create a coating that can be applied in one stage. PTFE was chosen by us because it describes a possible application of PTFE in the food industry. In particular, PTFE has shown itself well as a material for reducing adhesion to the working surfaces of units at milk processing plants [12,91]. It has also been shown that the adhesion of bacteria to working surfaces made of PTFE is reduced compared to other materials [92]. However, in our case, the pure PTFE did not have a bacteriostatic effect, which indicated that the antibacterial effect was realized through Fe_2_O_3_ NP-dependent mechanisms.

At the last stage of the study, the developed composite material was tested for its cytotoxicity against eukaryotic cells using mouse fibroblasts as an example (Figure 4 and Figure 5). A significant difference was found in the toxicity of the PTFE composite with 0.1% Fe_2_O_3_ NPs against bacteria (85% cell death) (Figure 6) and animal cells (no significant increase in the number of dead cells) (Figure 8a). Such a difference in toxicity could be explained by the more advanced structure of the repair and antioxidant defense systems in eukaryotic cells compared to prokaryotes [93,94,95,96]. Despite the absence of an increase in the number of dead cells, the PTFE/Fe_2_O_3_ NP composite material could not be called completely inert with respect to eukaryotic cells. An increase in the size of cell nuclei compared to the control with the PTFE without NPs was found (Figure 8b), which may indicate the onset of cell death processes and was consistent with the literature data on the cytotoxic effect of Fe_2_O_3_ NPs [97,98]. It should be noted that the literature data on the cytotoxicity of Fe_2_O_3_ NPs are ambiguous. On the one hand, some authors report a very low cytotoxicity of Fe_2_O_3_ NPs [66,99]. On the other hand, some works describe a clear cytotoxic effect of Fe_2_O_3_ NPs on epithelial cells (human lungs, HUVEC, hepatocytes, cell lines BEAS-2B and A549, etc.) due to the development of "oxidative stress", protein aggregation, impaired glucose metabolism, the excessive activation of the PI3K/Art-signaling pathway, and Caspase-3-dependent apoptosis [100,101,102,103,104]. The toxicity of Fe_2_O_3_ NPs in vivo has also been reported, with a predominance of kidney and liver damage [100]. Our results indicated a very low cytotoxicity of Fe_2_O_3_ NPs (Figure 8a); however, it may increase with prolonged use of the material, which was indirectly evidenced by an increase in the size of nuclei (Figure 8b). The received data about the count of dead cells (Figure 8) were consistent with the literature data about the good biocompatibility of Fe_2_O_3_ NPs. The search for the optimal concentration of Fe_2_O_3_ NPs and/or a new type of conjugates for NPs (glycol, proteins (BSA), or peptides (Arg-Gly-Asp) [105,106,107]) to ensure a balance between the high bacteriostatic activity of the developed composite material and the absence of toxicity against eukaryotic cells is a task for further research in this direction.

## 5. Conclusions

A new coating based on PTFE and Fe_2_O_3_ NPs was successfully developed in this study. The obtained coating had high adhesion to cutting boards made from PTFE and demonstrated a high surface quality (no defects at the microscopic level). The PTFE/Fe_2_O_3_ NP coating increased the generation of H_2_O_2_, 8-oxoguanine, and LRPS. The effect of the composite coating on reactive species generation depended on dose of Fe_2_O_3_ NPs in the PTFE matrix. The PTFE/Fe_2_O_3_ NP composites with NP concentrations of 0.001–0.1% inhibited bacterial growth, wherein the PTFE/Fe_2_O_3_ NPs (0.1% NPs) had no cytotoxicity against animal cells. These properties make coatings based on PTFE and Fe_2_O_3_ NPs a promising candidate as a protective coating for cutting boards in the meat processing industry.

## Figures and Tables

**Table 1:** The literature data on the antibacterial activity of composites of nanoparticles and polymer matrixes.

№	Synthesis Method	Composition	Size	MIC	Microorganism	Effect	Ref
1	Laser ablation in dimethyl formamide or sodium dodecyl sulphate	α-Fe_2_O_3_	50–110	4.25 mg/mL	*E. coli,* *P. aeruginosa,* *S. aureus,* *S. marcescens*	BS ^1^	[78]
2	Co-precipitation method	Fe_2_O_3_	25–40	10–50 µg/mL	*E. coli,* *S. aureus,* *S. dysentery*	BS	[79]
3	Co-precipitation method	Fe_2_O_3_	~50	0.5 mg/mL	*B. subtilis,* *E. coli,* *P. aeruginosa,* *S. aureus*	BS	[80]
4	Wet chemical method	Fe_3_O_4_	33–40	25–100 µg/mL	*E. coli,* *P. vulgaris,* *S. aureus,* *Xanthomonas* sp.	BS	[81]
5	Co-precipitation method	Fe_3_O_4_	6–9	32–128 μg/mL	*E. coli,* *L. monocytogenes,* *P. aeruginosa,* *S. marcescens*	BS	[82]
6	Co-precipitation method	Fe_3_O_4_	10.64 ± 4.73	50–500 µg/mL	*E. coli,* *E. hirae*	BS	[60]
7	Co-precipitation method	α-Fe_2_O_3_, ZnO/α-Fe_2_O_3_	~30	400–800 µg/mL	*B. subtilis,* *E. coli,* *S. aureus,* *S. typhimurium*	BS	[84]
8	Co-precipitation method	Fe_2_O_3_, FeO, coated with gentamicin	10–15	200 µg/mL	*B. subtilis,* *E. coli,* *P. aeruginosa,* *S. aureus*	BC ^2^	[85]
10	Co-precipitation method	Fe_3_O_4_ coated with chitozan	~11	30–40 μg/mL	*A. niger,* *B. subtilis,* *C. albicans,* *E. coli,* *F. solani*	BS	[86]
11	Laser ablation in water	Fe_2_O_3_ NPs/carbon nanotubes	6–7	400–800 μg/mL	*E. coli,* *K. pneumonia,* *S. aureus*	BS	[87]
12	Electrolysis	Ag NPs/PTFE	150	~5 mg/mL	*E. coli,* *S. aureus*	BC	[88]
13	Electrolysis + sol–gel method	Ag NPs/PTFE	500	~50 mg/mL	*E. coli*	BC	[27]
14	Laser ablation in water	Fe_2_O_3_/PLGA	50 nm	10 μg/mL	*E. coli*	BS	[83]
15	Laser ablation in water	Fe_2_O_3_/PTFE	60 nm	10 μg/mL	*E. coli*	BS	Current study

^1^ BS—bacteriostatic activity; ^2^ BC—bactericidal activity.

## Data Availability

The raw data supporting the conclusions of this article will be made available by the authors, without undue reservation.
